# The Risk of Developing Constipation After Neonatal Necrotizing Enterocolitis

**DOI:** 10.3389/fped.2020.00120

**Published:** 2020-04-03

**Authors:** Shan-Ming Chen, Jing-Yang Huang, Ming-Chi Wu, Jia-Yuh Chen

**Affiliations:** ^1^Department of Pediatrics, Chung Shan Medical University Hospital, Taichung, Taiwan; ^2^Department of Pediatrics, School of Medicine, Chung Shan Medical University, Taichung, Taiwan; ^3^Institute of Medicine, Chung Shan Medical University, Taichung, Taiwan; ^4^Department of Medical Research, Chung Shan Medical University Hospital, Taichung, Taiwan; ^5^Department of Medical Imaging, Chung Shan Medical University Hospital, Taichung, Taiwan; ^6^Department of Medical Informatics, Chung Shan Medical University, Taichung, Taiwan; ^7^School of Medicine, Chung Shan Medical University, Taichung, Taiwan; ^8^Department of Pediatrics, Changhua Christian Children's Hospital, Changhua, Taiwan

**Keywords:** adjusted hazard ratios, constipation, dysbiosis, failure to thrive, necrotizing enterocolitis

## Abstract

**Background:** Neonatal necrotizing enterocolitis (NEC) is a complex and lethal inflammatory bowel necrosis that primarily affects premature infants. Gut dysbiosis has been implicated in the pathogenesis of NEC. We aim to assess the association between NEC and two other diseases in children, including allergic diseases and constipation, considered to be associated with the alterations in gut microbiota composition.

**Methods:** This retrospective population-based cohort study was conducted using the Taiwan Birth Registration Database, Birth Certificate Application, and National Health Insurance Research Database to inter-link the medical claims of neonates and their mothers. A total of 2,650,634 delivery events were retrieved from 2005 to 2015. We identified a NEC cohort and selected a comparison cohort according to propensity score matching (1:1). Cox proportional hazard regression models were used to determine possible associations of predictors and to obtain adjusted hazard ratios (aHRs).

**Results:** A total of 1,145 subjects in the NEC cohort and 1,145 subjects in the matched cohort were analyzed during the observation period. No significant difference was observed in the incidence of allergic diseases between the two groups. NEC patients had a significant 30.7% increased risk of developing constipation (aHR = 1.307; 95% CI 1.089–1.568). The cumulative incidence of constipation was significantly higher in the NEC cohort than in the matched cohort by the end of follow-up (log-rank test *P* = 0.003).

**Conclusion:** Infants with NEC have a significantly higher incidence rate of developing constipation and FTT but no increased risk of allergic diseases.

## Introduction

Neonatal necrotizing enterocolitis (NEC) is a serious inflammatory intestinal disease in newborns, and is mostly seen in premature and low birth weight infants. Despite the advances in neonatal care over the past few decades, it remains a challenging condition in neonatal intensive care units because of significant mortality and long-term neuro-developmental morbidity (1). The reported mortality rate of NEC ranges from 18 to 63% (2). The pathogenesis of NEC is poorly understood but is likely to be multifactorial, including genetic susceptibility, intestinal immaturity, unbalanced micro-vascular tone, abnormal intestinal microbial colonization, and immuno-reactive intestinal mucosa (3). Advances in next generation sequencing technologies have revolutionized molecular biology research and increased understanding of the role of gut microbiota in the pathogenesis of certain diseases. Dysbiosis of the intestinal microbiota has been linked to pediatric diseases including NEC, asthma and atopic diseases, inflammatory bowel disease, and obesity (4). In addition, constipation is one of the most common childhood disorders, accounting for 10–25% of pediatric gastroenterology visits (5, 6). The pathophysiology of constipation in children is not fully understood and evidence suggests that the intestinal microbiota may play a role in the pathogenesis of functional constipation (7, 8). However, there is limited research on the relationship between these diseases and associations with early gut dysbiosis.

The aim of the present study was to explore the risk of subsequently developing constipation and allergic diseases in infants with NEC. The possibility of chronic malnutrition was also assessed in order to provide clues for pediatricians to begin prompt nutritional recommendations and interventions.

## Patients and Methods

### Data Source

**Figure 1:**
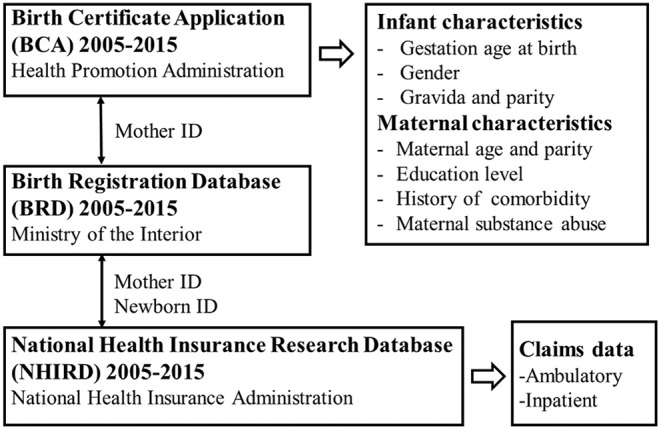
Flow chart of data processing between the three databases.

This study used three different nationwide population-based databases to inter-link the medical claims of neonates and their mothers (Figure 1). The first was the Birth Certificate Application (BCA, time frame from 2005 to 2015), which was retrieved from the Health Promotion Administration of the Ministry of Health and Welfare, Taiwan. The data covered all live births in Taiwan and stillbirths with a gestational age of more than 20 weeks or a birth weight of more than 500 g. This database included basic information about maternal background (nationality, place of residence, educational level), pregnancy status (maternal parity, maternal disease history, or special treatment), neonatal data (birth weight, gestational age, congenital defects), and maternal spouse information. The second database was the Birth Registration Database (BRD, time frame from 2005 to 2015), which was retrieved from the Ministry of the Interior, Taiwan. This database provided basic demographic data including national identification number, gender, date of birth, place of birth, parent's identification number, marital status, education level, and occupation. The BRD plays an important role in linking data with the other two databases. The third database was the National Health Insurance Research Database (NHIRD, time frame from 2005 to 2015), which was retrieved from the National Health Insurance Administration, Taiwan. The National Health Insurance (NHI) program is a compulsory health-care system launched in 1995 and currently covers more than 99% of the 23 million inhabitants of Taiwan and 93% of the country's clinics and hospitals. The NHIRD contains complete ambulatory and inpatient care claims data from all NHI enrollees.

### Ethical Statement

Data on the national identification card numbers of all subjects required for data linkage were encrypted. Data from the three databases were linked by the Health Welfare Data Science Center (HWDC) of the Ministry of Health and Welfare, Taiwan to facilitate the process and protect patient privacy. Only the results of statistical analysis for research purposes without personal information were released after careful inspection by HWDC staff. This study design was approved by the hospital's Institutional Review Board (CSMUH CS17044).

### Study Design and Population

**Figure 2:**
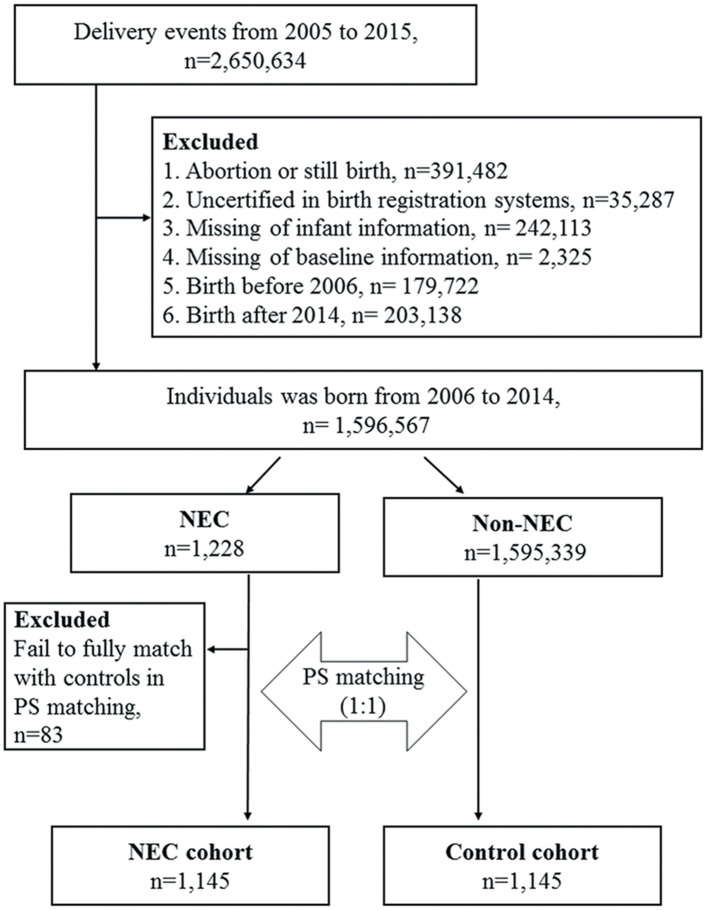
Flowchart of the patient selection process.

A total of 2,650,634 delivery events from 2005 to 2015 were retrieved from the linkage of the BCA 2005–2015 and BRD 2005–2015 by examining the common characteristics including birth date, birth weight, sex, gestational age of the newborns, mother's identification number and birth date, and father's birth date. To minimize potential information bias, we excluded cases of abortion or stillbirth, those without full certification between the two birth registration databases, and those with missing infant or baseline data. We also excluded those with a birth date before 2006 and after 2014 on account of insufficient maternal baseline characteristics in the databases and a short follow-up duration. These results were then linked externally to the NHIRD according to the identification numbers of the newborns and their mothers to extract data for this retrospective cohort analysis, including an NEC cohort and a comparison group. The selection process of the study subjects and comparison cohort from the databases is shown in Figure 2. NEC patients were defined as those with an admission with an International Classification of Diseases, 9th revision, Clinical Modification (ICD-9-CM) code for NEC (777.50 unspecified, 777.51 Stage I, 777.52 Stage II, 777.53 stage III) within 60 days from the date of birth. The stage of NEC was defined according to modified bell's staging criteria (9). Stage I NEC is associated with mild intestinal or systemic signs, non-specific or normal radiological signs. Stage II NEC is characterized by gastrointestinal signs, specific radiological signs (pneumatosis intestinalis or portal venous gas), and abnormal laboratory findings (metabolic acidosisor thrombocytopenia). Stage III NEC includes patients with severe systemic illness, DIC and, severe radiological signs (pneumoperitoneum). The index date for the patients with NEC was defined as the date of first hospitalization, and the index date of the control subjects was defined as the same index date as the paired NEC patient. The subsequent development of allergic and gastrointestinal outcomes of the enrolled subjects with NEC were evaluated, including atopic dermatitis (ICD-9-CM code 691), allergic rhinitis (ICD-9-CM code 477), asthma (ICD-9-CM code 493), constipation (ICD-9-CM code 564.0), failure to thrive (FTT) (ICD-9-CM code 783.41). Patent ductus arteriosus (PDA) (ICD-9-CM code 747.0), Intraventricular hemorrhage(IVH) (ICD-9-CM code 772.1–772.14). To ensure accurate coding of these diagnoses, only patients with at least one inpatient claims record or three or more ambulatory claims with the same diagnosis were included. Patients with onset of asthma were identified after 2 years of age to avoid diagnostic uncertainty.

### Propensity Score Matching

Propensity score matching is a statistical procedure used to reduce potential bias and baseline differences to allow for a more reasonable comparison between study and control groups. In this study, we used 1:1 matching in propensity score to obtain an appropriate control cohort. Propensity scores were calculated using a logistic regression model based on covariates including index year, sex, multiple-births, birth order, gestational age, birth weight of the newborn and age, education level, and maternal disease.

### Statistical Analysis

Differences between the study and control groups regarding the characteristics of the infants and mothers were compared using the chi-square test. To evaluate the risk of subsequent NEC-related comorbidities, Cox proportional hazard models were used to estimate the hazard ratios (HRs) and 95% confidence intervals (CIs) in the NEC and comparison cohorts during the follow-up period. The cumulative incidence rates for constipation, and FTT in both groups were plotted using Kaplan-Meier analysis, and differences between the two cohorts were examined using the log-rank test. All data were recorded and analyzed using SAS Statistics software (version 9.4; SAS Institute, Inc., Cary, NC, USA). The follow-up period was calculated in months using claims data and shown as median and interquartile range (IQR). A *P* < 0.05 was considered to be statistically significant.

## Results

A total of 1,145 NEC patients and 1,145 matched control subjects were selected for the study (Table 1). Both the NEC and matched cohorts contained more males than females and exhibited a similar distribution of infant and maternal characteristics. Although 82.36% of the babies who developed NEC were premature, 202 (17.64%) term infants also developed the disease. Table 2 presents the incidence and adjusted HRs (aHRs) of diseases between the NEC cohort and matched controls. There were no statistically significant differences in the incidence of asthma, allergic rhinitis, or atopic dermatitis between the two groups. A total of 262 (22.9%) of NEC patients presenting with constipation (262 vs. 211, *P* = 0.008) and 59 (5.2%) NEC patients had FTT (59 vs. 30, *P* = 0.002). Compared with the matched controls, the patients with NEC had a significantly higher incidence rate of constipation and FTT, especially in the male patients. The aHRs were 1.307 (95% CI 1.089–1.568) for developing constipation and 2.073 (95% CI 1.329–3.233) for developing FTT in the NEC patients vs. the matched controls.

**Table 1:** Baseline characteristics of the NEC cohort and matched controls.

**Characteristic**	**Controls (*n* = 1,145) *n* (%)**	**NEC (*n* = 1,145)*n* (%)**	***P-*value**	**Standardized difference**
**Infant characteristics**				
Year of birth			1.000	0.043
2006	123 (10.74%)	123 (10.74%)		
2007	140 (12.23%)	142 (12.40%)		
2008	145 (12.66%)	143 (12.49%)		
2009	123 (10.74%)	122 (10.66%)		
2010	108 (9.43%)	109 (9.52%)		
2011	120 (10.48%)	120 (10.48%)		
2012	133 (11.62%)	133 (11.62%)	
2013	117 (10.22%)	116 (10.13%)		
2014	136 (11.88%)	137 (11.97%)		
Sex			0.966	<0.001
Male	597 (52.14%)	598 (52.23%)		
Female	548 (47.86%)	547 (47.77%)		
Multiple-births			0.852	−0.046
No	854 (74.59%)	83 1 (72.58%)		
Yes	291 (25.41%)	31 4 (27.42%)		
Birth order			0.705	−0.016
1	640 (55.90%)	631 (55.11%)		
≥ 2	505 (44.10%)	514 (44.89%)		
Gestational age at birth (weeks)			0.728	0.026
<24	12 (1.05%)	11 (0.96%)		
24–36	917 (80.09%)	932 (81.40%)		
≥37	216 (18.86%)	202 (17.64%)		
Birth weight (g)			0.777	0.029
<1,500	494 (43.14%)	501 (43.76%)		
1,500–2,499	431 (37.64%)	434 (37.90%)		
2,500–3,499	194 (16.94%)	179 (15.63%)		
≥3,500	26 (2.27%)	31 (2.71%)		
Delivery method			0.113	−0.066
Vaginal delivery	464 (40.52%)	427 (37.29%)		
Cesarean section	681 (59.48%)	718 (62.71%)		
PDA	74(6.46%)	150(13.1%)	<0.001	0.225
IVH	21(1.83%)	44(3.84%)	0.004	0.121
**Maternal characteristics**				
Maternal age (Mean±SD)	30.9 ± 4.8	31.1 ± 4.9	0.923	0.093
10–19	15 (1.31%)	21 (1.83%)		
20–24	87 (7.60%)	84 (7.34%)		
25–29	296 (25.85%)	297 (25.94%)		
30–34	481 (42.01%)	469 (40.96%)		
35–39	221 (19.30%)	225 (19.65%)		
≥40	45 (3.93%)	49 (4.28%)		
Education level			0.810	0.046
Junior high school or lower	61 (5.33%)	71 (6.20%)		
Senior high school	457 (39.91%)	446 (38.95%)		
College	547 (47.77%)	551 (48.12%)		
Master or above	80 (6.99%)	77 (6.72%)		
Maternal disease history				
Hypertension	149 (13.01%)	138 (12.05%)	0.488	−0.029
DM	68 (5.94%)	78 (6.81%)	0.392	0.036
Renal disease	18 (1.57%)	15 (1.31%)	0.599	−0.022
Genitourinary infection	94 (8.21%)	98 (8.56%)	0.763	0.013
General infection	18 (1.57%)	21 (1.83%)	0.628	0.020
Anemia	119 (10.39%)	113 (9.87%)	0.678	−0.017
Mental disorder	37 (3.23%)	41 (3.58%)	0.645	0.019
Asthma	56 (4.89%)	59 (5.15%)	0.774	0.012
Allergic rhinitis	259 (22.62%)	255 (22.27%)	0.841	−0.008

*DM, diabetes mellitus; IVH, intraventricular hemorrhage;PDA, patent ductus arteriosus; SD, standard Deviation*.

**Table 2:** Incidence and adjusted hazard ratios of subsequent disease stratified by sex between the NEC cohort and matched controls.

	**Controls (*****n*** **=** **1,145)**			**NEC (*****n*** **=** **1,145)**			**Crude HR (95% CI)**	**Adjusted HR (95% CI)**
	**Events**	**PM**	**Rate**	**Events**	**PM**	**Rate**		
Asthma	277	58,709	4.72	242	580,662	4.17	0.881 (0.742–1.047)	0.882 (0.742–1.049)
Male	166	29,730	5.58	152	28,928	5.25	0.930 (0.746–1.159)	0.873 (0.734–1.039)
Female	111	28,979	3.83	90	29,138	3.09	0.811 (0.614–1.071)	0.794 (0.599–1.052)
Allergic rhinitis	389	50,234	7.74	381	50,450	7.55	0.975 (0.846–1.123)	0.968 (0.840–1.116)
Male	218	25,677	8.49	219	25,145	8.71	1.009 (0.836–1.217)	1.008 (0.834–1.219)
Female	171	24,557	6.96	162	25,305	6.40	0.936 (0.755–1.160)	0.925 (0.744–1.150)
Atopic dermatitis	377	48,653	7.75	347	48,073	7.22	0.919 (0.794-1.063)	0.923 (0.797–1.069)
Male	187	26,129	7.16	188	24,000	7.83	1.022 (0.835–1.251)	1.000 (0.815–1.227)
Female	190	22,524	8.44	159	24,073	6.60	0.818 (0.662–1.009)	0.834 (0.673–1.033)
Constipation	211	58,331	3.62	262	53,919	4.86	1.309 (1.092–1.569)	1.307 (1.089–1.568)
Male	105	31,160	3.37	127	28,307	4.49	1.292 (0.998–1.673)	1.321 (1.017–1.717)
Female	106	27,171	3.90	135	25,612	5.27	1.320 (1.023–1.702)	1.280 (0.989–1.657)
Failure to thrive	30	68,790	0.44	59	65,285	0.90	2.044 (1.317–3.172)	2.073 (1.329–3.233)
Male	12	36,284	0.33	30	34,265	0.88	2.635 (1.349–5.147)	2.649 (1.349–5.201)
Female	18	32,506	0.55	29	31,020	0.39	1.656 (0.920–2.981)	1.542 (0.847–2.807)

*Rate: per 1,000 person months*.

*Adjusted HR: multivariable analysis including sex, multiple-births, birth order, gestational age and birthweight*.

*CI, confidence interval; HR, hazard ratio; NEC, necrotizing enterocolitis; PM, person-months*.

**Figure 3:**
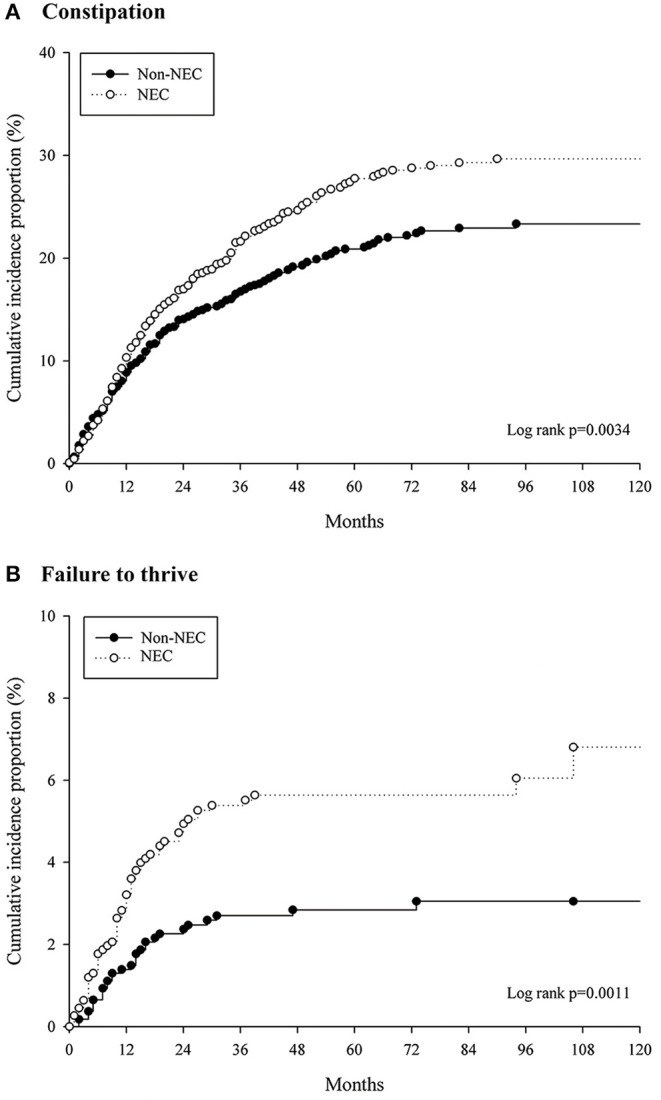
Cumulative incidences of **(A)** constipation and **(B)** failure to thrive events for the patient with NEC and matched controls.

Long-term hospitalization (>60 days) occurred in 26.8% of NEC stage I+ II and 50% of NEC stage III (*P* < 0.001). The aHR of FTT was 3.408 (95% CI 1.939–5.988) in the NEC stage III patients with a compared to NEC stage I+ II (reference) (Table 3). The median follow-up time was 41 months (IQR 16–78 months) in the patients with constipation and 56 months (IQR 25–89 months) for those with FTT. Kaplan-Meier survival analysis showed that the cumulative incidence of constipation and FTT events were significantly higher in the NEC group (log rank *P* = 0.003 and log rank *P* = 0.001, respectively) (Figure 3).

**Table 3:** Risk of constipation and failure to thrive at different stage of NEC.

**Incidence rate**	**NEC (*****n*** **=** **1,145)**		**Adjusted HR (95% CI)**
	**Stage I+II (*n* = 975) (reference)**	**Stage III (*n* = 170)**	
Constipation	4.88	4.68	0.812 (0.544–1.213)
Failure to thrive	0.64	3.36	3.408 (1.939–5.988)

*Rate: per 1,000 person months*.

*CI, confidence interval; HR, hazard ratio; NEC, necrotizing enterocolitis*.

## Discussion

Dysbiosis is considered to be an alteration in the function or structure of the microbiome, and is typically characterized by an expansion of pathobionts, loss of beneficial commensals and loss of microbial diversity (10). In particular, gut microbial dysbiosis has been implicated in several pediatric diseases, including NEC (4). Several lines of evidence support the association between NEC and intestinal microbiota composition (11–14). In addition, experimental NEC has not been reproduced in germ-free animals, and NEC rarely develops until at least 8–10 days after birth in preterm neonates, the period when intestinal anaerobic bacterial colonization begins (3). However, it is still not entirely clear whether gut dysbiosis is a cause or consequence of NEC, an issue compounded by the complexity of this disease. By using the unique advantages of the NHIRD, including a large population and long-term follow-up, we conducted this population-based retrospective cohort study to examine the preliminary risk of developing allergic diseases and constipation in infants presenting with NEC in early life.

“Atopic march” describes the sequential progression of allergic symptoms from atopic dermatitis during infancy to asthma and allergic rhinitis in early life (15). Although atopic dermatitis usually occurs between 3 and 6 months of age, the onset of symptoms may also start at or before 2 months, the period approximately when NEC occurs (16). Some studies have reported the use of oral supplementations with probiotics in the treatment of atopic dermatitis, and their findings seem to indirectly strengthen the association between gut dysbiosis and the development of atopic dermatitis (17, 18). Our results showed no significant increase in the risk of developing atopic dermatitis, allergic rhinitis, and asthma in the NEC patients (median follow-up times of 29, 37, 43 months, respectively). This could mean that early dysbiosis of gut microbiota may not be the only mechanism leading to allergic diseases. Besides host genetics and environmental factors, gut microbiota-derived metabolites, disturbances in the skin or lung microbiome may play more crucial modulatory roles in the development of allergic diseases.

Previous research has indicated that <10% of children with constipation are a result of organic diseases such as Hirschsprung's disease, anorectal malformations, spinal cord anomalies, endocrine or metabolic disorders (19, 20). In the majority of cases, there is no obvious organic explanation. Similar to other functional gastrointestinal disorders, the cause of functional constipation is considered to be multifactorial, and a low fiber diet, painful defecation with stool withholding, and slow colonic transit have been implicated (21). Recent evidence suggests that alterations in gut microbiota and pelvic floor dysfunction through the “microbiota-gut-brain axis” appear to play an essential role in the etiology of constipation (8, 22, 23). In addition, a recent study indicated that gut microbiota can affect gastrointestinal motility through microbiota-derived short-chain fatty acids and various mediators such as gut hormones or cytokines (24).

Our results showed that the patients in NEC group were prone to develop PDA and IVH, even though the proportion of preterm births was similar between the NEC group and matched controls. PDA, hypoxia, and hypotension have been identified as risk factors for NEC and IVH (25, 26), and these factors are commonly responsible for circulatory instability or ischemic and hypoxic injury. IVH is considered to be a predictor of neurodevelopmental outcomes in preterm infants (27). However, due to the retrospective design of this study design, our findings cannot be used to infer a causal relationship between PDA or IVH and constipation. In addition, the patients with stage III NEC were not prone to have constipation but had a 240.8% increased risk of FTT compared to the stage I+II patients. That is, mild to moderate intestinal inflammation in early life may be enough to cause a certain degree of damage to intestinal motility. Higher severity of disease, greater treatment intensity, and longer hospital stay may have contributed to suboptimal catch-up growth in these stage III patients. Given that stage I NEC may be clinically similar to sepsis or septic ileus, we further used the code for stage II NEC (777.52) and excluded those who were prescribed with antibiotics for less than 7 days and those with any intestinal perforation, and identified 416 patients who met stage II NEC. The stage II NEC patients had a significant 29.5% increased risk of developing constipation (aHR = 1.295; 95% CI 1.018–1.646) compared to the matched controls. This is similar to the results of the three-stage overall analysis.

Previous studies have shown microscopic evidence of the pathologic changes occurring in NEC, including a lack of sympathetic innervation of submucous arteries, no or few synaptic nerves in the myenteric plexus of the bowel wall, risk of leukocytic infiltration of the muscularis propria, and atrophic desmosis in the muscularis propria by leukocyte collagenases (28). The absence of synaptic nerves in the myenteric plexus can lead to increased spasticity of the colon, which can then narrow the bowel lumen and retard the forward movement of stools. Focal atrophic desmosis of the colon has been observed in pediatric patients resulting from inflammation processes in the muscularis propria. Clinically, such changes in the bowel wall are thought to contribute to constipation with a hypoperistalsis syndrome (28, 29). Nevertheless, longitudinal studies with a large sample size to support the above pathological inference are lacking. This study reports NEC patients were more susceptible to constipation. It suggests that pathologic changes occurring in NEC may cause long-term intestinal functional effects. However, there is currently insufficient evidence to support gut dysbiosis in early life as a major factor contributing to the development of constipation. Future prospective cohort studies are needed to prove that causal link. In gender-stratified analysis, the risk in girls did not reach significance, there was still a positive trend for constipation. Gender specific differences in the prevalence of child constipation have yet to be verified (21). In general, girls usually develop most toileting skills and successfully achieve toilet training earlier than boys (30, 31). Further research is needed to clarify this issue.

Due to the need for long-term follow-up, the intestinal function of NEC patients after treatment has rarely been studied, and large reference materials are limited to date. The current study provides some implications for the gastrointestinal care of NEC patients after hospital discharge. The first 1,000 days (from conception to 2 years of age) is the most critical window for growth and development of children, and it is essential to monitor the growth velocity and provide sufficient dietary calories, especially for patients with stage III NEC. Moreover, it is necessary to take prompt and appropriate action, including promoting prolonged breastfeeding, regular dietary fiber recommendations, and timely toddler toilet training, especially for male patients.

There are several strengths to this study. Linking three nationwide databases minimized the impact of information errors and yielded comprehensive data on maternal characteristics. The NHIRD covers nearly the entire population of Taiwan with minimal selection bias, long duration of follow-up, and multi-institutional follow-up linkage. There are also some limitations to this study. First, the NHIRD is composed of all enrollees' complete ambulatory and inpatient care claims data, which allows researchers to trace the use of all medical services by the enrollees. However, physical examination findings, laboratory test results, the etiology of disease, and type of feeding are not provided in the claims data of NHIRD. Second, the validity of diagnostic codes might be questioned. In order to minimize diagnostic coding errors, only subjects with at least one inpatient claims record or three or more ambulatory claims were recruited. Our advanced analysis revealed that 92.1% of the NEC patients were from tertiary medical centers or metropolitan hospitals, and such institutes are certain to have qualified neonatal specialists and neonatal intensive care services. Hence, their diagnoses can be considered to be reliable. In addition, a previous study has already proven a moderate concordance between the claims records in the NHIRD and self-reported survey results with regards to clinical diagnoses (32). Third, propensity score matching was used to attempt to obtain a well-matched group so as to remove potential bias due to observed confounders. However, some unobserved confounding factors can still bias the results. Food and medications could potentially contribute to constipation in young children. Similarly, maternal allergic disease, lack of breast-feeding, and early exposure to antibiotics may affect childhood allergic outcomes. Using the second control group has often been mentioned to examine the impact of unobserved biases. Compared with a second comparison group composed of enrollees from the same population and observation period as the study group, the incidence of constipation and FTT events were still remained significantly higher in the NEC group (aHRs 1.225, 95% CI 1.024–1.466 and aHRs 1.800, 95% CI 1.171–2.766, respectively).

In conclusion, infants with NEC have a significantly higher incidence rate of developing constipation and FTT but no increased risk of allergic diseases. More prospective research is required to determine the roles of early gut dysbiosis in the long-term effect on intestinal motility and catch-up growth.

## Data Availability Statement

The datasets for this article are not publicly available because: the data analyzed in this study was obtained from Health Welfare Data Science Center (HWDC) of the Ministry of Health and Welfare, Taiwan. Requests to access the datasets should be directed to [HWDC, hcrdc@tmu.edu.tw].

## Ethics Statement

The studies involving human participants were reviewed and approved by the Institutional Review Board/Ethics Committee, Chung Shan Medical University Hospital. Written informed consent from the participants' legal guardian/next of kin was not required to participate in this study in accordance with the national legislation and the institutional requirements.

## Author Contributions

S-MC and J-YC devised study conception and the project. J-YH and J-YC obtained funding and revised the initial draft of manuscript. S-MC, J-YH and M-CW performed acquisition of data, statistical analysis and interpretation of data. S-MC drafted the manuscript. J-YC supervised the project. All authors have approved the final submitted version.

### Conflict of Interest

The authors declare that the research was conducted in the absence of any commercial or financial relationships that could be construed as a potential conflict of interest.
